# Adult-Onset Annular Pancreas: When to Intervene?

**DOI:** 10.7759/cureus.67728

**Published:** 2024-08-25

**Authors:** Iqbal M Ali, Vijay Sai Reddy M, Varun Shetty

**Affiliations:** 1 General Surgery, Dr. D. Y. Patil Medical College, Hospital and Research Centre, Dr. D. Y. Patil Vidyapeeth, Pune, IND

**Keywords:** autopsy, diagnosis, incidentally, congenital abnormality, annular pancreas

## Abstract

Annular pancreas in adults is a rare congenital abnormality, often detected after the onset of complications or incidentally during autopsy. Diagnosis in adults is challenging due to the similarity of symptoms with other conditions. We report a case of a 55-year-old female who presented with a six-month history of intermittent colicky pain in the epigastric region, radiating to the back, accompanied by periodic nonbilious vomiting. Abdominal examination revealed mild tympany and succussion splash in the epigastric region, with no organomegaly, lumps, or visible peristalsis. The patient was treated successfully with gastrojejunostomy (GJ) and jejunojejunostomy (JJ), leading to a good recovery. This case highlights the importance of considering the annular pancreas in the differential diagnosis of adults presenting with similar symptoms, underscoring the diagnostic challenges faced by radiologists and surgeons.

## Introduction

Annular pancreas, a rare congenital defect, is defined by the presence of pancreatic tissue encircling the second part of the duodenum. This tissue, varying in width, can cause partial or complete obstruction of the duodenum. It is estimated that this condition occurs in approximately 1 out of every 12,000-15,000 live births. The prevalence of annular pancreas in adults, as determined through autopsy cases, has been reported to range from 0.005% to 0.015% [1].

During the fourth week of gestation, the ventral and dorsal pancreatic buds arise from the junction of foregut and midgut. The ventral pancreatic bud is initially paired (right and left ventral pancreatic buds). However, subsequently, the left ventral pancreatic bud regresses during the development of pancreas. The development of pancreas occurs as a result of the side to side fusion of the ventral and dorsal pancreatic buds during the seventh week of gestation [2]. The ventral pancreatic bud forms part of the head, bod, and tail of the pancreas. The uncinated process and a part of the head of pancreas develops from the ventral pancreatic bud. The main pancreatic duct is created by the fusion of the ventral pancreatic duct in the head region and the lower portion of the dorsal pancreatic duct in the body and tail regions. The accessory pancreatic duct is derived from the dorsal pancreatic duct during embryonic development. The annular pancreas arises when the ventral pancreatic bud fails to migrate properly during embryonic development, leading to the encirclement of the duodenum by pancreatic tissue [2]. This condition can manifest with a broad range of symptoms and varying degrees of severity. While it is typically diagnosed in newborns, its presentation in adults can be quite diverse, often mimicking other gastrointestinal conditions [3].

The formation of an annular pancreas is primarily attributed to theories by Leeco and Baldwin. Leeco’s theory suggests that the condition arises when the ventral pancreatic bud adheres to the duodenal wall during embryonic development, preventing its normal migration. This results in the formation of a ring of pancreatic tissue around the duodenum, typically involving the second part (D2) in about 74% of cases. This theory could be explained by the presence of more abundant pancreatic polypeptide (PP)-laden islet cells in the ventral pancreatic bud. Baldwin’s theory suggested that the occurrence of annular pancreas is secondary to the persistence of the left ventral pancreatic bud, which usually regresses by the sixth week of gestation [3].

In adults, the clinical presentation of the annular pancreas can include symptoms that are easily confused with other disorders, such as acid peptic disease, acute or chronic pancreatitis, and dysfunction of the sphincter of Oddi [4]. This variability in presentation contributes to the diagnostic challenge, as the symptoms overlap significantly with these more common conditions. Consequently, patients may undergo extensive evaluations and treatments for these alternative diagnoses before the true cause of their symptoms is identified. The diagnostic complexity is further compounded by the fact that the annular pancreas is relatively rare, leading to a lower index of suspicion among clinicians. Furthermore, the occurrence of annular pancreas in adults can be easily confused with other differentials, such as primary duodenal malignancies, extrinsic compression by tumors arising from the pancreatic head, or even gastrointestinal tuberculosis (TB) (especially in TB-laden countries like India).

A variety of diagnostic tools can be employed to identify the annular pancreas, including imaging modalities such as abdominal ultrasound, computed tomography (CT) scans, magnetic resonance imaging (MRI), and endoscopic retrograde cholangiopancreatography (ERCP) [5]. However, each of these techniques has its limitations and may not definitively diagnose the condition in all cases. For instance, while CT and MRI can provide detailed images of the pancreatic tissue and its relation to the duodenum, they may not always reveal the extent of the encirclement. ERCP, though more invasive, can offer a more direct visualization of the pancreatic ducts but carries higher risks. Given these diagnostic challenges, a high degree of clinical suspicion is necessary, and a combination of investigative modalities is often required. We present a case of symptomatic annular pancreas in an elderly female, illustrating the diagnostic journey and therapeutic intervention that led to a successful outcome.

## Case presentation

A 64-year-old female patient presented with a history of nonbilious vomiting episodes and intermittent pain in the upper abdomen for the last six months. The vomiting occurred predominantly postprandial, which raised concerns about the underlying cause. The patient reported a history of one to two episodes of vomiting daily. The abdominal pain was colicky in nature and was aggravated post-meals. General physical examination was largely unremarkable, with vital signs falling within normal limits. The abdominal examination did not reveal organomegaly, providing minimal diagnostic insight. The classical signs of gastric outlet obstruction, including a positive succussion splash were absent. Routine biochemical and hematological investigations returned normal results (Table 1), providing no immediate clues to her symptoms.

**Table 1:** Laboratory parameters (preoperative) with normal laboratory ranges SGOT: Serum glutamic-oxaloacetic transaminase; SGPT: serum glutamate pyruvate transaminase; CRP: C-reactive protein; TLC: total leukocyte count

Investigation	Observed parameters	Reference interval (as per our institutional laboratory)
Total bilirubin	1.1 mg/dl	0.22-1.20 mg/dl
Conjugated bilirubin	0.7 mg/dl	Upto 0.5 mg/dl
Unconjugated bilirubin	0.4 mg/dl	0.1-1.0 mg/dl
SGOT	42 U/L	8-48U/L
SGPT	40 U/L	7-55U/L
Alkaline Phosphatase	100 U/L	40-129U/L
CRP	2.5 mg/L	Upto 5.0 mg/L
Urea	40 mg/dl	17-49 mg/dl
Serum creatinine	1.1 mg/dl	0.6-1.3 mg/dl
Hemoglobin (Hb)	13.2 g/dl	13.2-16.6mg/dl
TLC	6800/ uL	4000-10000/ul
Serum amylase	42 U/L	40-140 U/L
Serum lipase	40 U/L	50-120 U/L

An upper gastrointestinal endoscopy revealed an insignificant benign ulcer at the pyloric region. However, the endoscopic evaluation could not be extended past the second part of the duodenum (D2) due to a narrowing caused by extrinsic compression and resulting proximal dilatation of the duodenum. This limitation prompted further imaging studies to clarify the etiology of the obstruction.

**Figure 1:**
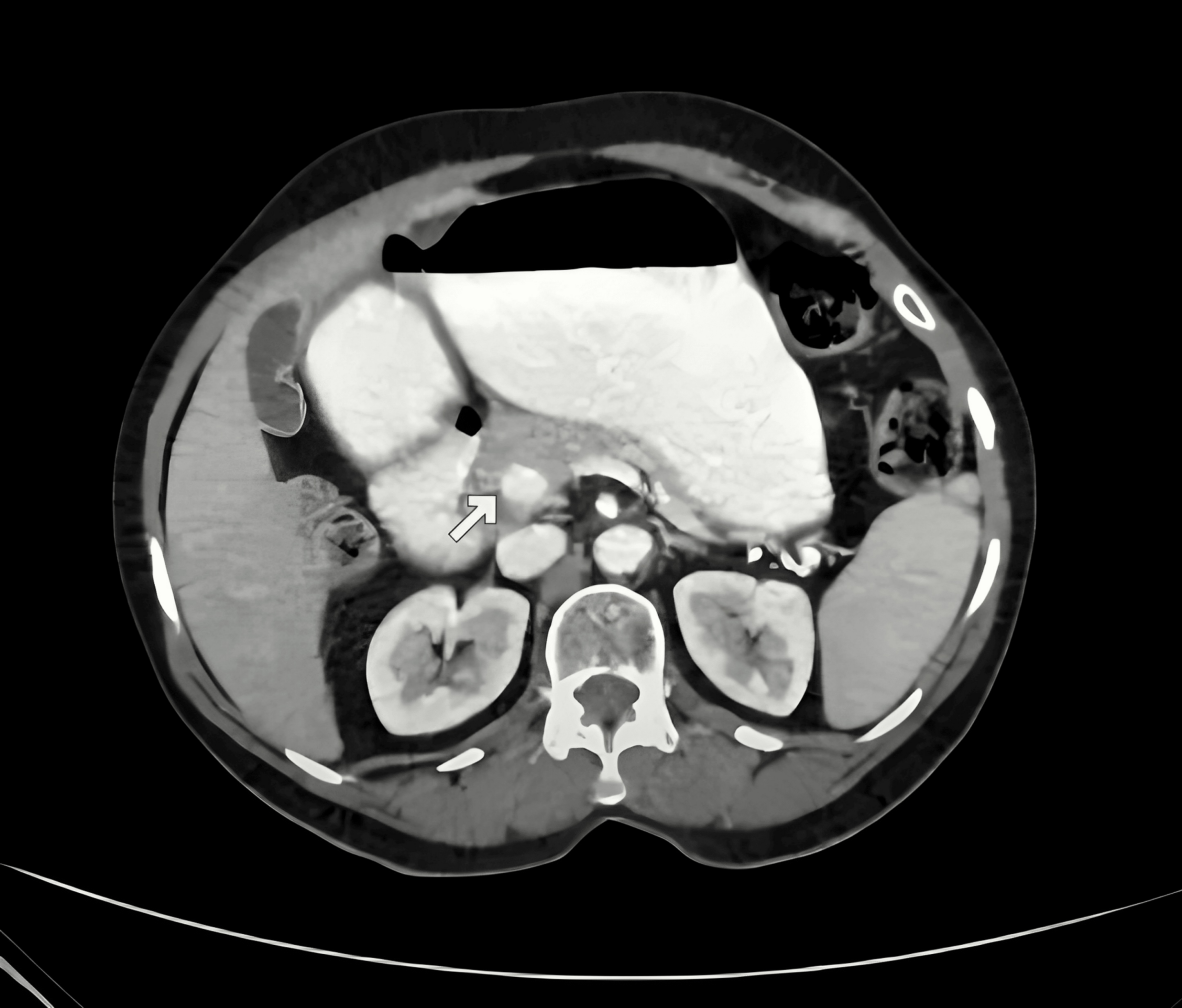
CECT image (presurgery) demonstrating annular pancreas surrounding the 2nd part of the duodenum CECT: Contrast-enhanced computed tomography -White arrow indicates the annular ring of the pancreatic parenchyma surrounding the 2nd part of duodenum

**Figure 2:**
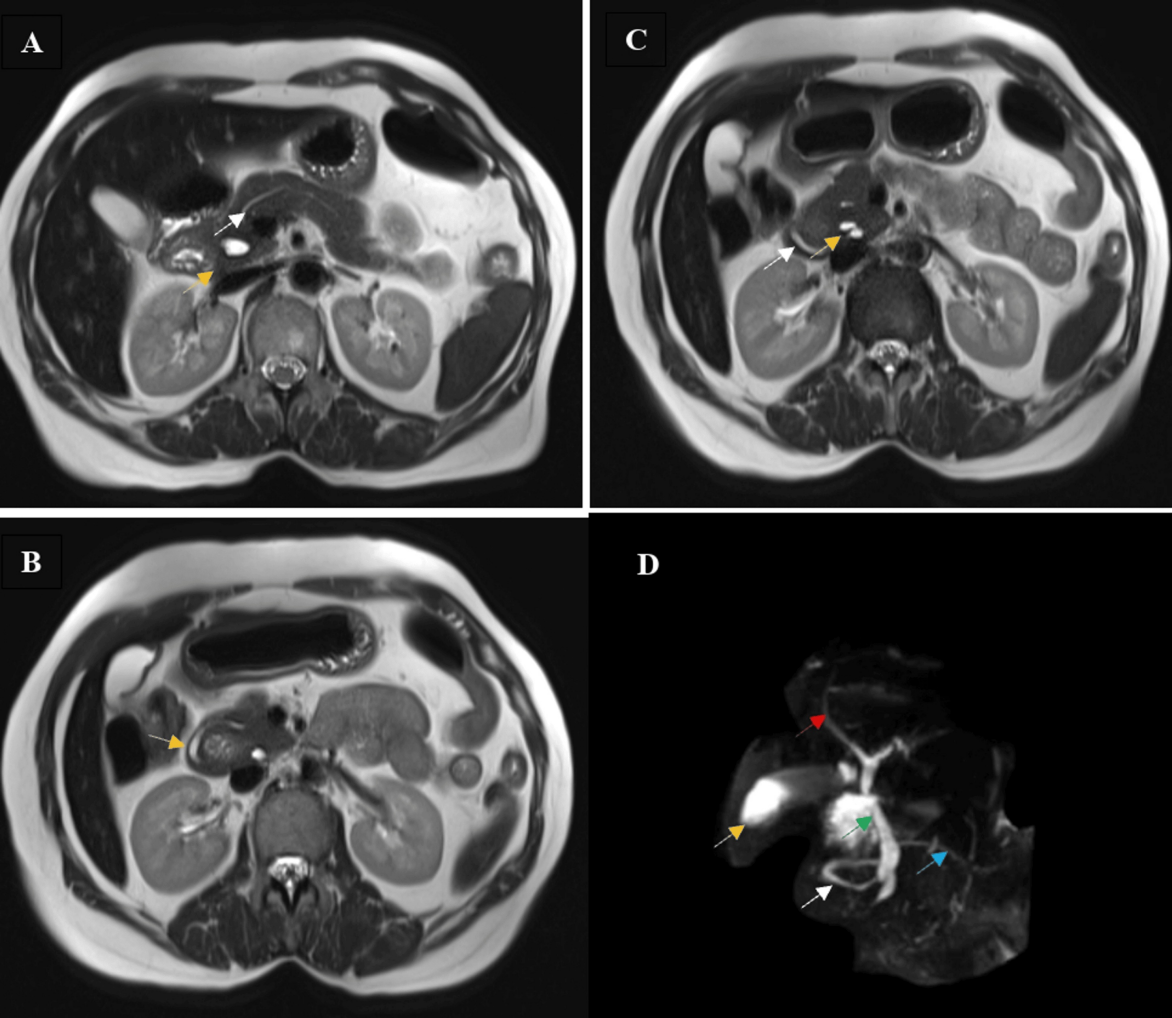
Serial MRCP films showing annular pancreas (A-D) MRCP: Magnetic resonance cholangiopancreatography (A) MRCP (T2 image/transverse cut): White arrow indicates the main pancreatic duct (MPD); yellow arrow indicates the encircling pancreatic parenchyma. (B) MRCP (T2 image/transverse cut): Yellow arrow indicates the MPD encircling the duodenum. (C) MRCP (T2 image/transverse cut): White arrow indicates the relation of MPD in relation to the common bile duct (CBD) indicated by the yellow arrow. (D) Reconstructed MRCP image showing the annular pancreas where the red arrow indicates right hepatic duct (RHD), yellow arrow indicates the gall bladder, green arrow indicates the common bile duct (CBD), white arrow indicates the annular pancreatic duct, and blue arrow indicates the MPD

A contrast-enhanced CT (CECT) scan of the abdomen showed a thin segment of pancreatic tissue encircling the second part of the duodenum (Figure 1). Magnetic resonance cholangiopancreatography (MRCP) was performed to better understand the ductal architecture and the extent of the pancreatic involvement. This imaging study confirmed the pancreatic duct and a thin segment of pancreatic tissue around the second part of the duodenum, causing mild inflammation and compression, which explained the observed duodenal narrowing (Figure 2). This thin rim of pancreatic tissue encircling the duodenum resulted in duodenal obstruction due to extrinsic compression. These findings were consistent with a typical presentation of annular pancreas, especially in adults.

**Figure 3:**
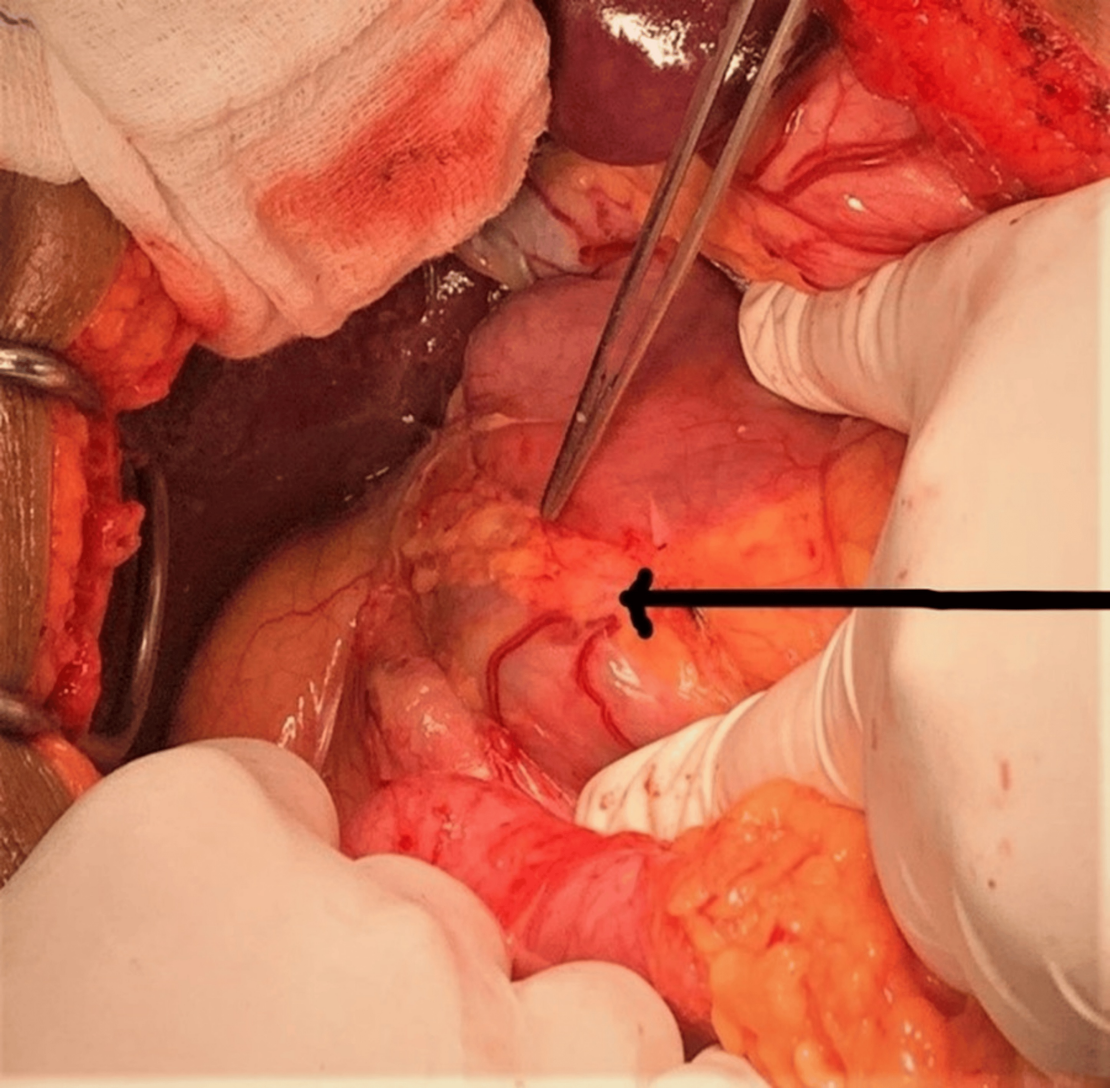
Intraoperative image The black arrow shows a thin rim of pancreatic tissue encircling the 2nd part of duodenum, resulting in proximal duodenal dilatation

Subsequently, an exploratory laparotomy was conducted. During the procedure, it was observed that the first part of the duodenum was dilated, and the second part was surrounded by a 2 cm-wide band of pancreatic tissue, which was responsible for the proximal duodenal dilatation (Figure 3). To address this anatomical anomaly and relieve the obstruction, an anterior highly selective vagotomy and isoperistaltic retrocolic posterior gastrojejunostomy (GJ) were performed.

The patient’s postoperative course was uneventful, and she was discharged on the 12th postoperative day. This case underscores the importance of detailed imaging in diagnosing and managing unusual gastrointestinal obstructions and illustrates the effectiveness of surgical intervention in resolving such complex issues.

## Discussion

Pancreatic fusion abnormalities, including the annular pancreas and pancreatic divisum, have varying prevalences and presentations. Among these, the annular pancreas is notably rare in adults, with prevalence estimates in India ranging between 15 and 400 cases per 100,000 adults [6]. The condition is exceedingly uncommon in adults, with historical evidence such as Vasconcelos and Sadek's report of one instance out of 22,243 autopsies highlighting its rarity [7]. However, the true prevalence may be underreported due to the limited frequency of duodenal dissection during autopsies. Recent studies, such as one by Karasaki et al., identified an incidence of 1.14% based on institutional CT scan assessments, indicating a higher frequency due to improved imaging techniques [8]. In rare instances, it may be associated with cancer or obstructive jaundice [9].

Advances in radiological imaging have increased the detection rate of the annular pancreas in adults, from 0.2% in 2000 to 2.1% in 2021 [10]. Despite these advancements, more than 40% of diagnoses still require surgical confirmation due to the limitations of imaging modalities. For instance, CECT scans may be limited by the narrow ring structure, while ERCP may be invasive and complicated by pancreatitis or lumen narrowing. MRCP is also restricted by its requirement for a dilated ductal system.

Management of annular pancreas typically necessitates surgical intervention when symptoms are present. For patients with duodenal obstruction, enteric diversion surgery is often performed, with duodeno-duodenostomy being preferred due to its minimal creation of blind loops and more physiological outcomes [11]. Alternatively, GJ, which can lead to stoma ulcerations, may require a second vagotomy in younger patients. If inflammation and adhesions in the C loop are present, duodenojejunostomy may not be feasible. Resection of pancreatic parenchyma, while documented, is generally reserved for cases where cancer cannot be excluded due to its potential for severe complications, including local inflammatory and fibrotic reactions [11]. Unlike children who undergo duodenal bypass procedures, Zyromski et al. emphasized the intricate pancreaticobiliary pathology linked to acute pancreatitis in adults. They reported that 20% of adult patients in their study group who needed surgical intervention underwent complex pancreaticobiliary surgical procedures such as pancreaticoduodenectomy, lateral pancreatojejunostomy, and biliary/pancreatic sphincteroplasty [12]. Despite its low incidence, the presence of gastric outlet obstruction and abdominal pain in adults should prompt consideration of an annular pancreas, with treatment and surgical approaches tailored to individual cases. The potential association with malignancy necessitates careful evaluation to rule out cancer.

## Conclusions

This case emphasizes the critical role of comprehensive imaging studies in identifying and diagnosing unusual presentations of an annular pancreas. Despite normal biochemical and hematological results, imaging techniques like CT and MRI/MRCP were essential in revealing the pancreatic tissue encircling the duodenum, causing the obstruction. The surgical intervention, including an anterior highly selective vagotomy and isoperistaltic retrocolic posterior GJ, effectively alleviated the obstruction and led to an uneventful recovery. This demonstrates that detailed imaging and appropriate surgical management are vital for successfully treating complex gastrointestinal cases, ultimately enhancing patient outcomes.
